# Ventral Caudate and Anterior Insula Recruitment During Value Estimation of Passionate Rewarding Cues

**DOI:** 10.3389/fnins.2020.00678

**Published:** 2020-07-29

**Authors:** Isabel Catarina Duarte, Gonçalo Coelho, Sónia Brito-Costa, Ricardo Cayolla, Sónia Afonso, Miguel Castelo-Branco

**Affiliations:** ^1^Coimbra Institute for Biomedical Imaging and Translational Research, Institute for Nuclear Sciences Applied to Health, Faculty of Medicine, University of Coimbra, Coimbra, Portugal; ^2^Human Potential Development Center, Institute of Applied Research, Polytechnic Institute of Coimbra, Coimbra, Portugal; ^3^Department of Economics, Management, Industrial Engineering and Tourism, University of Aveiro, Aveiro, Portugal; ^4^Porto Business School, University of Porto, Porto, Portugal

**Keywords:** reward, decision making, ingroup, striatum, insula, caudate

## Abstract

“Wanting”, a component of reward processing, is a motivational property that guides decision making in goal-oriented behavior. This includes behavior aiming at supporting relational bonds, even at the group level. Accordingly, group belongingness works as this motivational property, which is fundamentally different from romantic or maternal love. While primary rewards (or learned associations, such as money) have been largely used to study the conceptual framework associated with “wanting,” other cues triggering behavior, such as passionate motives, are less well-studied. We investigated the neural correlates of value estimation of a passion-driven incentive in neuropsychologically defined football fans. We asked the participants (*n* = 57) to compute the value of football tickets (the cues that trigger passionate behavior in this “tribal love” context). The trials were all different, comprising tickets for different matches. The participants had no restrictions on the amount to be introduced. This enabled a parametric functional magnetic resonance imaging design based on the explicit estimated value given by the participants in a trial-by-trial approach. Using a whole-brain approach (to prevent biased focus on value-related regions), only the activity in the ventral caudate and left anterior insula showed a critical relationship with the reported value. Higher normalized values led to more activity in the striatum and left insula. The parametric map shows that these regions encode the magnitude of incentive by indexing self-relevant value. Other regions were involved in value computation, such as the ventromedial prefrontal cortex, lateral orbitofrontal cortex, and dorsolateral prefrontal cortex, but did not exhibit parametric patterns. The involvement of the nucleus accumbens in value estimation was only found in region of interest -based analysis, which emphasizes the role of the ventral caudate for the presently studied social “reinforcer” cue.

## Introduction

Group belongingness supports relational bonds and represents a human need (Baumeister and Leary, 1995). Humans need to be connected to peers, nurturing ingroup relations. While romantic and maternal love represent a form of bonding between individuals with clear biological relevance for reproduction and survival, bonding and identification with unrelated members at the scale of a group are important in a broader social context. Self-preservation and safety motives may underlie this phenomenon (Brewer, 2007) such that belongingness feelings create a strong motivation to nurture social relationships (Baumeister and Leary, 1995). One piece of evidence for this is the well-known feature of human behavior: ingroup bias. The feeling of group belongingness is known to bias empathy and helping attitudes among diverse social groups (Molenberghs, 2013; Cikara and Van Bavel, 2014; Hackel et al., 2017; Molenberghs and Louis, 2018).

Neuroimaging studies of group belongingness-based decision making are rare. Sports fandom can be used as an ecological context to study group belongingness as a motivational factor (Bortolini et al., 2017) that guides decision making in goal-oriented behavior.

Generally, human goal-oriented behavior is characterized by seeking reward maximization and avoiding punishment (Tobler et al., 2007; Wallis, 2007), so decision making is critically dependent on the capacity of an organism to estimate contextual values (Yacubian et al., 2006). The expected (reward) value is computed when triggered by a cue, and it can be different from the reward value itself (Knutson et al., 2005). The reward value computation implies the experience of that eventual outcome itself. In this way, reward processing comprises multiple components, which include “wanting” and “liking” (Pool et al., 2016). “Wanting” and “liking” may cohere, but they can also be different (Zhang et al., 2009; Berridge, 2012; Pool et al., 2016).

The expected value signals the utility of an action, eliciting motivation, which guides decision making. Ultimately, the expected value triggered by a cue explains the amount of effort one is willing to invest to obtain it.

Activations in the orbitofrontal and ventromedial prefrontal cortices and in subcortical structures such as the ventral tegmental area have long been associated with the reward experience itself (Rogers et al., 1999; Elliott et al., 2000; O’Doherty et al., 2001; Small et al., 2001; Anderson et al., 2003; Gottfried et al., 2003; D’Ardenne et al., 2008). The ventromedial prefrontal cortex is thought to process the magnitude of the rewarding experience during the outcome phase (Knutson et al., 2000, 2005; Gottfried et al., 2003; Bray and O’Doherty, 2007; Diekhof et al., 2012). Nucleus accumbens activation has been associated with prediction error signaling (Knutson et al., 2001; Yacubian et al., 2006; D’Ardenne et al., 2008). As the mesolimbic dopamine system projects from the ventral tegmental area to the nucleus accumbens (among other structures, such as the amygdala, hippocampus, and orbitofrontal cortex), this pathway is considered to play a fundamental role in reward processing and signaling prediction error for reinforcement and learning mechanisms. As a core region in the ventral striatum, the involvement of the nucleus accumbens in reward processing is quite well established for mainly monetary rewards (Breiter et al., 2001; Knutson et al., 2001; Yacubian et al., 2006). For practical reasons, money is the reward cue type most used in neuroimaging studies. Nevertheless, others rewarding cues have confirmed the role of the nucleus accumbens in reward processing, for example, food (O’Doherty et al., 2002) and food odors (Gottfried et al., 2003), and even images with sexual content (Brand et al., 2016).

Sports fandom drives fans to engage in game-related socially rewarding activities. Our previous neurobehavioral study showed that passion for a football team has very strong affective properties, with neural recruitment of reward system areas while seeing related images (Duarte et al., 2017). Moreover, this functional magnetic resonance imaging (fMRI) study showed that activity in reward-related and limbic regions, such as the ventral tegmental area/substantia nigra, amygdala, putamen, pallidum, and insula, was higher for participants who scored higher in a sport fanaticism scale (Duarte et al., 2017).

For a fan, the opportunity to attend a sports game live may create a dilemma involving putting in money and effort for the sake of the reward. Such reward expectation often drives fans toward largely consumptive behavior that may sometimes seem inappropriate. We speculate that such value processing involving passion-related desires is quite distinct from value processing for food or goods, due to the nature of the cue. This is a decision-making process under some kind of emotional utility rather than the common utility assigned to a primary reinforcer (like food) or a secondary reinforcer (like money) for which rewarding value is acquired by learned associations with primary reinforcers (Balleine et al., 2007).

In this study, we asked whether value estimation of socially related cues recruits the same neural systems as that of primary appetitive cues. Due to the nature of the cue, we questioned whether the value computation involving group belongingness desires may be distinct from such a computation involving food or goods (i.e., cues intrinsically linked to survival). In the present work, we aim to understand the neural correlates of value estimation in the context of ingroup belongingness. To achieve that goal, we used sports fandom as an ecological context in which to study group belongingness as a motivational factor (Bortolini et al., 2017; Duarte et al., 2018). We asked 57 football fans to rate football match tickets during fMRI. The computation of the magnitude of such values could be studied using a parametric design. We created a parametric design where we forced the value estimation of the match tickets to be dynamically changed by incorporating into its computation different contextual sources of value (namely the teams playing, the existence of rivalry, the tournament, or the phase in the tournament).

## Materials and Methods

### Participants

Fifty-seven subjects completed behavioral and imaging experiments. The sample was composed of 55 males and two females, aged from 21 to 60 years, 34.7 ± 10.9 years (mean ± standard deviation, SD). Fifty-six out of 57 used the joystick in the right hand, given their handedness. All of the subjects had normal or corrected to normal vision.

The participants were fans of *Futebol Clube do Porto* (Porto) or *Associação Académica de Coimbra* (Académica) as characterized elsewhere (Duarte et al., 2017). The two teams were playing in the Portuguese First League at the time of the acquisitions. The group is the same cohort as in Duarte et al. (2017, 2018), and as described there, participants completed two assessments: one to assess personal identification with the team [Sport Spectator Identification Scale, SSIS (Wann and Branscombe, 1993; Theodorakis et al., 2010)] and another to assess fanaticism for football [Football Supporter Fanaticism Scale, FSFS (Wachelke et al., 2008)]; both used a 1–5 Likert scale. Note that in a same-day study (Duarte et al., 2017), activity in the ventral tegmental area/substantia nigra, amygdala, putamen, pallidum, and insula showed to be correlated with FSFS for nearly the same cohort of participants (55 participants shared between both studies).

All subjects signed a form expressing informed consent of this study, which was approved by the Ethics Committee of the Faculty of Medicine of the University of Coimbra, in accordance with the Declaration of Helsinki.

### Data Acquisition

The scanning session was performed in a 3T Magnetom Trio Tim MRI scanner (Siemens, Erlangen, Germany) using a 12-channel birdcage head coil. A T1-weighted MPRAGE anatomical volume was acquired with a repetition time (TR) of 2530 ms, echo time (TE) of 3.42 ms, resolution of 1 mm^3^, flip angle of 7°, matrix size of 256 × 256, field of view of 256 × 256, and slice thickness of 1 mm. To correct the functional images for geometrical distortion, we acquired gradient field maps (GRE) before each Echo Planar Imaging (EPI) sequence. Phase and magnitude field maps were acquired with TR of 3000 ms, TE of 30 ms, echo spacing of 0.5 ms, 100% phase resolution, phase encoding direction from anterior to posterior, echo time difference of 2.46 ms, and bandwidth in the phase direction of 31.25 Hz. Functional data were obtained using EPI sequences. The 170 volumes were acquired with a slice thickness of 3 mm and voxel size of 4 mm^2^, 36 slices acquired parallel to the AC-PC line, TR of 3000 ms, TE of 30 ms, flip angle of 90°, matrix size of 256 × 256, and FOV of 256 × 256.

Stimuli were displayed on an LCD monitor (NordicNeuroLab, Bergen, Norway) with a frequency rate of 60 Hz and dimensions of 69.84 cm × 39.29 cm that was placed approximately 156 cm away from the participant’s head. The subject could actively give responses using a magnetic resonance-compatible joystick (Hybridmojo, San Mateo, CA, United States).

### Task Design

Before the fMRI session, the participants were told that they would be asked for the value that they would offer to attend a specific match. We gave all participants the same instruction: “Imagine that you are already near the stadium at the time of the game and you do not have a ticket. You do not have to consider the extra costs of traveling. You find someone selling one ticket for the match; however, there is no room for negotiation. You only have one opportunity to make an offer and get the ticket. How much would you offer?” The instruction emphasized the personal value of that ticket and was therefore more dependent on its “emotional utility.” Before fMRI, the participants performed a training task with non-football related questions (e.g., How much would you offer for: a concert/your favorite band in your city), to get familiarized with the task timings and with joystick usage.

**FIGURE 1:**
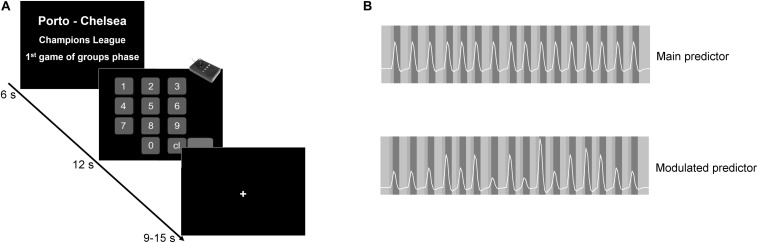
Experimental design and predictor models. **(A)** The paradigm had a boxcar design. In the first block, the participants appraised the match evaluation described by teams playing, tournament, and stage in the tournament. Participants were then asked to introduce the amount they were willing to pay to attend that game. Each match evaluation/response pair was followed by a jittered baseline. **(B)** Example of the main and the modulated predictors of one particular participant. The standard main contrast allows a search for main effects of the estimation value condition, while the parametric contrast allows a search for modulatory effects (regions in which activity increases as the value estimation increase).

Inside the scanner, the participant faced the value estimation task. The experiment has a boxcar design with two conditions in addition to the baseline. The first condition block (6 s) presented the match evaluation, and it was followed by the response block. The paradigm had 16 trials for match evaluation and 16 corresponding response blocks. The text presented on the match evaluation block was always different, always describing different matches. However, it was always defined by three components, as seen in Figure 1A: (1) the teams playing, (2) the tournament, and (3) the stage in the tournament. Twelve out of 16 matches involved the participant’s preferred team. The remaining four matches involved two small teams from Series E of the National Championship (high probability of being unknown, functioning as a non-rewarding condition). In the conditions involving the participant’s preferred team, the match could be against either a weaker team, a strong rival team, or a European team. The context (tournament) also varied, and it was described as, for example, National championship or Champion’s League. Similarly, we varied the stage in the tournament, such as, for example, “final” or “first match of the group phase.” By changing the “playing teams,” the “tournament,” and the “phase in the tournament” components, we dynamically incorporated variable sources of value into the computation of the estimated value for each match. The matches were presented randomly, so the order of presentation was different for all participants. Each match evaluation block was followed by the response block, where the participant provided his/her estimated value. This value was used later to create the parametric modulator. Each one of the 16 match evaluation/response pairs was followed by a baseline lasting between 9 and 15 s (see Figure 1A). The participant had no restrictions concerning the amount to be introduced.

### Data Analysis

Pre-processing and analysis were done using BrainVoyager QX 2.8.2 (Brain Innovation, Maastricht, Netherlands). Functional images were corrected for geometrical distortions using the AnatAbacus v1.1 plugin (Breman et al., 2009) for BrainVoyager QX. Data were corrected for: (1) slice scanning time differences using cubic spline interpolation; (2) motion, combining trilinear- and sinc-function-based methods for interpolation in the three axes; and (3) filtered in the time domain using a GLM (general linear model) approach with a Fourier basis set with two cycles per time course. The anatomical and functional data were co-registered (and manually verified) and then normalized according to the Talairach atlas. After the spatial normalization, spatial smoothing was performed using a Gaussian kernel of 4 mm FWHM (full width at half maximum). A mask was obtained by averaging all functional files, excluding bone, scalp, eyes, and cerebellum.

A parametric analysis was performed to isolate effects related to the magnitude of the estimation by assigning a weight to each trial. The weight assigned to each trial was defined as the value introduced by the participant in each response block. The response blocks were not considered for the fMRI analysis. These weights were intra-individually normalized between 0 and 1. According to this procedure, differences in the individual incomes of the participants do not affect the overall group results (concerning socioeconomic status, only 39 out of 57 participants responded to the income question in the report form). Two predictors were created: (1) the main predictor, a standard predictor, in which the boxcar function (with value 1) is convolved with a two-gamma hemodynamic response function (Figure 1B – top panel), and (2) the modulated predictor, calculated similarly to the previous, but in which the boxcar function has a variable value according to the weight in each trial. Here, the parametric weight differentially modulates the boxcar function trial by trial according to the estimated value (Figure 1B – bottom panel). Additionally, by performing a conjunction analysis, we increase the specificity of the design, ensuring that a region responding to the parametric modulation also responds to the main contrast. In this way, the main contrast compares the match evaluation block versus the baseline, but it is not used in isolation but rather in conjunction with a parametric contrast to ensure specificity (Cohen, 1997; Price and Friston, 1997; Friston et al., 1999). The conjunction analysis works as an extension to the contrasts, and it considers only the commonality in those contrasts. Here, by considering as parametrically responsive areas only those regions that had shown to be involved in the task (main contrast), the conjunction analysis allows control for false positives (Cohen, 1997; Price and Friston, 1997; Friston et al., 1999). On the other hand, the parametric contrast will give us the areas in which the activity level is related to the weight. So, the higher the activity, the higher the amount introduced by the participant. This parametric approach takes into account the continuous nature of ticket ratings and has a larger statistical power than simple categorical “high vs. low” comparisons.

General linear model random effects (RFX) analysis was performed at the group level with a focus on the decision period prior to the report. The RFX-GLM statistical maps were corrected for multiple comparisons using Bonferroni correction for the main contrast. For the parametric approach, after the conjunction analysis, the map was corrected using cluster threshold levels with a *p*-value of 0.01 and a voxel extent of 31, the estimation of which was based on Monte Carlo simulations (1000 iterations).

The main purpose of statistical inference was to perform a parametric analysis using a whole-brain approach based on the behavioral data of each participant. However, to help with further discussion of the role of other areas that are known to be involved in reward value estimation, namely, the nucleus accumbens and ventromedial prefrontal cortex (vmPFC), we performed an region of interest (ROI)-based analysis. In this, we defined ROIs on the nucleus accumbens, vmPFC, and ventral caudate. While the vmPFC was functionally defined from the main contrast, the ventral caudate was functionally defined from the parametric contrast (here just to allow for comparisons). The nucleus accumbens was anatomically defined. We ran an ROI-GLM on the three ROIs individually with *p* set at 0.05 and corrected using FDR (false discovery rate).

## Results

### Behavioral Results

The maximum value offered to see a football match was €500, the minimum was €0, and the average of the individuals’ averages was €18.22 ± 13.39 (grand mean ± SD). The average of the individual maximum value was €87.09 ± 107.35, while the average of the minimum was €1.18 ± 1.77. The percentage of responses greater than zero was 87.68%. All participants gave the maximum value to a ticket of their favorite team for either the final of the national cup, the last game of the national championship, or the final of a European tournament.

Concerning fandom and team identification scales, one out of 57 volunteers did not provide answers for all items in both scales, while another one had missing values in the FSFS scale. Concerning the remaining responses, the identification scale (SSIS, mean score and SD = 4.10 ± 0.74, *n* = 56) and the fanaticism scale (FSFS, 3.16 ± 0.96, *n* = 55) were highly correlated [*r*(53) = 0.80, *p* < 0.00001].

### General RFX Analysis

**FIGURE 2:**
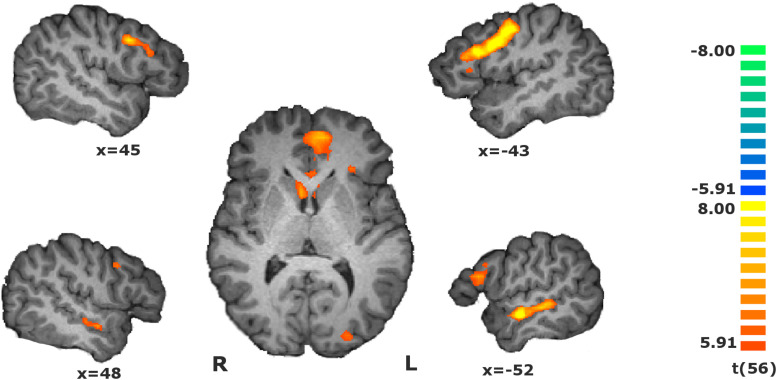
Statistical map of the main contrasts during the value estimation, i.e., during match evaluation block vs. baseline [RFX, *t*(56) = 5.91, *p* < 0.01, Bonferroni-corrected]. Activity increased in the dorsolateral and ventromedial prefrontal cortices, orbitofrontal cortex, insula, medial frontal gyrus, superior temporal sulcus, right inferior parietal lobule, and visual cortex. The map calculated at group level is overlain on a single-subject anatomical file for visualization purposes.

Investigation of the regions involved in value estimation, given by the main contrast (RFX, *t*(56) = 5.91, *p* < 0.01, Bonferroni-corrected) identified a cluster compromising the vmPFC, left lateral orbitofrontal gyrus, caudate head and left insula, and other clusters in the dorsolateral prefrontal cortex, right lateral orbitofrontal cortex and right insula, superior temporal sulcus, medial frontal gyrus and visual cortex, and in the left anterior insula. Negative changes were found in the inferior parietal lobule. The statistical map is presented in Figure 2, and the details are given in Table 1.

**TABLE 1:** Regions revealed by the main contrast during the match evaluation, i.e., during match evaluation [RFX, *t*(56) = 5.91, *p* < 0.01, Bonferroni-corrected].

		Tal coords					
	H	*x*	*y*	*z*	*t*	*p*	mm^3^
Dorsolateral prefrontal cortex	R	42	5	31	8.88	<0.000001	3074
Dorsolateral prefrontal cortex	L	−42	5	34	9.27	<0.000001	10483
vmPFC, caudate, left insula and lateral OFC	R,L	−6	38	−2	8.78	<0.000001	10478
Right lateral OFC and insula	R	30	32	4	7.32	<0.000001	1058
Medial frontal gyrus	R,L	−6	2	52	10.21	<0.000001	3983
Superior temporal sulcus	R	48	−7	−11	7.04	<0.000001	818
Superior temporal sulcus	L	−54	−10	−8	9.20	<0.000001	3318
Inferior parietal lobule	R	57	−31	31	−7.85	<0.000001	798
Visual cortex	R	21	−88	−8	6.76	<0.000001	364
Visual cortex	L	−12	−79	−8	9.79	<0.000001	5951

*The clusters are described by their hemisphere (H), peak voxel coordinates in the Talairach space, the *t*- and *p*-values in the peak voxel, and the number of voxels (in mm^3^).*

**FIGURE 3:**
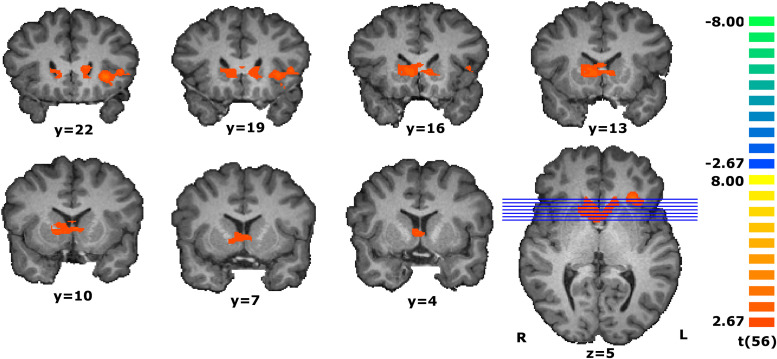
Coronal slices of the statistical map resultant from the conjunction analysis of the parametric contrast modulated by the estimated value and the main contrast during the match evaluation [RFX, *t*(56) = 2.67, *p* < 0.01, cluster extension-corrected, minimum cluster size of 31 contiguous voxels]. Activity in the ventral caudate and left insula increases as the estimated value increases. The map calculated at group level is overlain on a single-subject anatomical file for visualization purposes.

To discover the areas responding proportionally to the individual estimated values, we perform a parametrical analysis during the match evaluation phase by assigning a weight to each trial given by the participant’s response (see details in the data analysis subsection of the Materials and Methods). To increase specificity, we performed a conjunction analysis with two contrasts to find the regions presenting a modulatory effect. The conjunction analysis of the parametric contrast of value estimation and the main orthogonal contrast (RFX, *t*(56) = 2.67, *p* < 0.01, cluster extension-corrected) revealed that a ventral part of caudate nucleus and the left anterior insula presented a parametric effect concomitant with the stimulus’ magnitude (see Figure 3 and Table 2).

**TABLE 2:** Regions revealed by the conjunction analysis of the parametric contrast and the main contrast during the match evaluation [RFX, *t*(56) = 2.67, *p* < 0.01, cluster-corrected].

		Tal coords					
	H	*x*	*y*	*z*	*t*	*p*	mm^3^
Ventral caudate	R,L	15	11	4	3.80	0.000354	3595
Insula	L	−27	23	4	4.51	0.000034	1331

*The clusters are described by their hemisphere (H), peak voxels’ coordinates in the Talairach space, the *t*- and *p*-values in the peak voxel, and the number of voxels (in mm^3^).*

**FIGURE 4:**
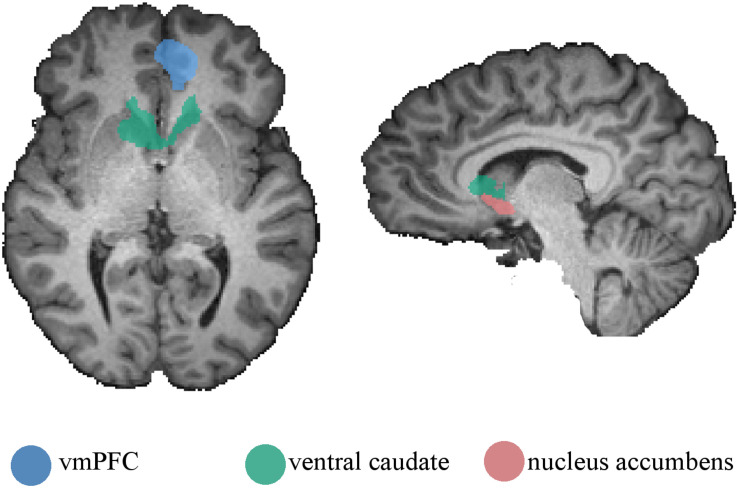
Regions of interest used on the ROI-based parametric analysis. Ventromedial prefrontal cortex, ventral caudate, and nucleus accumbens were defined from the main contrast, the parametric contrast, and anatomically, respectively. Regions are presented on a single-subject anatomical file for visualization purposes.

Given that other regions, such as the nucleus accumbens and the vmPFC, are involved in the processing of reward significance, we further conducted an ROI-based analysis. We ran ROI-GLM on the vmPFC, ventral caudate (functionally defined from the parametric contrast, here just to allow for comparisons), and the nucleus accumbens. The parametric contrast was performed on the three ROIs (RFX, *p* < 0.05, corrected using FDR). For the vmPFC, no voxel was activated, showing no parametric modulation by the estimated value. However, for the nucleus accumbens, two clusters appeared in the right [peak voxel with *t*(56) = 3.75, *p* = 0.00042] and left [peak voxel with *t*(56) = 3.44, *p* = 0.00112] accumbens. As expected from the prior whole-brain analysis, the ventral caudate again showed significant activations [peak voxel with *t*(56) = 3.80, *p* = 0.00035). Figure 4 depicts the ROIs in the nucleus accumbens, vmPFC, and ventral caudate.

## Discussion

Here, we studied the processing of value estimation in the context of ingroup belongingness. The participants, football fans, were asked to compute the effort – given by the ascribed value of a match ticket – they were willing to make to see a relevant football match. This value changed dynamically because the participant always faced different sources of value such as: teams playing, rivalries, tournament, and phase in the tournament. As the participant had no restrictions concerning the amount to be introduced, we could quantify the value magnitude using a parametric design. The values introduced by the participants were intra-individually normalized. Using a whole-brain GLM approach, we found a critical role for the ventral caudate in the computation of such magnitude properties: the higher the activity in ventral caudate, the higher the estimated value. Importantly, the left anterior insula similarly exhibited a parametric effect.

### Neural Correlates of the Value Estimation

Other reward and cognitive control-related areas were also activated during the value estimation but did not show evidence for parametric modulation by that value magnitude. These regions included the medial prefrontal cortex, lateral orbitofrontal cortex, and dorsal lateral prefrontal cortex. The type of value estimation studied here was quite unique due to involving an ingroup belongingness-related cue that was experimentally established in previous studies (Bortolini et al., 2017; Duarte et al., 2017, 2018). The estimation of a trade-off between the expected hedonic value and the monetary cost, reflecting some kind of emotional decision utility, is quite present in the life of the selected participants, who often make similar decisions on a weekly basis. We expect that these results can be extended to other social domains involving the estimation of value triggered by other ingroup belongingness drivers (for example, political affiliation-related drives or even ethnicity- and race-related motivations).

### Parametric Representation of the Value

The neural correlates of reward processing are quite well established in the literature by studies using mainly food, drink, or money triggers. Those studies generally identify the orbitofrontal cortex, ventromedial prefrontal cortex, anterior insula, and also subcortical structures such as the nucleus accumbens, the amygdala, and the substantia nigra, as well as the ventral tegmental area (Delgado et al., 2000; Elliott et al., 2000; Knutson et al., 2000; Berns et al., 2001; Breiter et al., 2001; Small et al., 2001; O’Doherty et al., 2002; Anderson et al., 2003). The extension to the social domain has been considered using reward stimuli such as beautiful faces (Aharon et al., 2001; Bray and O’Doherty, 2007) or mutual cooperation in playing the Prisoner’s Dilemma (Rilling et al., 2002).

Our parametric design allowed a weight to be attributed to each trial according to the participant’s response. The predictor was modeled according to those weights on a trial-by-trial basis. Instead of a *high vs. low* contrast, we performed a parametric analysis using a whole-brain approach, with no *a priori* ROI selection. This allowed us to show that activity in the ventral caudate and the left anterior insula changes parametrically in accordance with value magnitude: the higher the activity, the higher the computed value later introduced by the participant.

The role of reward magnitude in modulating neuronal activity in different parts of the primate striatum was previously demonstrated for different types of primary reward. For example, the magnitude of the quantity of reward juice was related to the single-cell discharge rate in monkey (Cromwell and Schultz, 2003). Here we provide evidence that, for more complex forms of reward, functional specialization may emerge within the striatum. In our findings, the striatal involvement comes mainly (or entirely) from the ventral caudate. Like the nucleus accumbens, the ventral caudate receives projections from dopaminergic neurons in the midbrain (Wise and Rompre, 1989; Schultz, 1998; Düzel et al., 2009). However, a major role in positive reinforcement of reward processing has been attributed to the nucleus accumbens (Aharon et al., 2001; Schultz, 2002; Floresco, 2015). Nevertheless, the dorsal striatum has also been suggested to be involved in motivational and learning processes that support goal-directed behavior (Balleine et al., 2007). Evidence from animal and human studies suggests that it encodes action-outcome associations in goal-directed behavior (Balleine et al., 2007).

The major role attributed to the nucleus accumbens could make one think that it should also play a major role in cues combining belongingness feelings in football-related activities for this cohort of participants. After the whole-brain parametric analysis, we performed an ROI-based analysis in nucleus accumbens (and also vmPFC) to search for a parametric modulatory effect from the value estimation. We found bilateral voxels showing this effect. Unlike the ventral caudate region, this parametric effect was only identified in the ROI-based approach. Results similar to ours, i.e., caudate instead of nucleus accumbens recruitment, were also reported for other reward-related cues, such as mutual cooperation (Rilling et al., 2002), faces (Bray and O’Doherty, 2007), and drugs (Cox et al., 2009). These findings provide generalization to the evidence that reward-related behavior and action-outcome evaluation specifically involves the ventral caudate (Pauli et al., 2016).

The parametric activation found in insula in the present task may be related to its role in the incentive salience computation. Incentive salience is a motivational property that may be elicited by reward-predicting cues (McClure et al., 2004; Berridge, 2012). The neural correlates of incentive salience are quite well established in the literature by studies using mainly drug and food triggers, which, generally, identify the anterior insula and the anterior cingulate cortex - regions defining the salience network - and also subcortical structures such as the amygdala and the ventral striatum (Berridge, 2012).

The fact that ventral caudate and anterior insula are parametrically activated as a function of the estimated value does not imply that these areas themselves are computing that value; however, it does suggest a strong involvement in the integration of this information for subsequent decision making.

### The Role of the Ventromedial Prefrontal Cortex

In neuroeconomic studies, decision making implicating reward value and prediction error processing has been largely related to the ventral striatum (nucleus accumbens) and ventromedial prefrontal cortex/orbitofrontal cortex (Knutson and Cooper, 2005; Knutson et al., 2005; Tobler et al., 2007; Mccabe and Redoute, 2008; Staudinger et al., 2009; Declerck et al., 2012). The ventromedial prefrontal cortex (including the medial part of the orbitofrontal cortex) has been commonly associated with the encoding of reward value (Diekhof et al., 2012). Ultimately, the reward value expectation defines the amount of effort one is willing to expend to obtain it. Accordingly, we were particularly interested in determining whether the ventromedial prefrontal cortex would exhibit a parametric modulation according to value magnitude, but this was not the case, as shown by the whole-brain parametric approach. To confirm this, we made a conventional ROI basis analysis in the vmPFC, functionally defined (from the main contrast). We categorized the participants’ responses into four levels of value. Although this was not the aim of this work, it allowed for a comparison with results in the literature, which are commonly reported as coming from a *high vs. low* contrast. The percentage of BOLD change in the level-based approach is quite similar among levels in the vmPFC. This provides evidence that, although vmPFC is involved in the value estimation of these triggers, it may not be directly involved in the computation of that value. That parametric effect, a possible signature of a neural computation of salience, was only found in the ventral caudate and left anterior insula. A meta-analysis suggests that the ventral striatum is also involved in anticipation of reward while the ventromedial prefrontal cortex processes the information mainly during the outcome phase (Diekhof et al., 2012) during the hedonic experience itself. In sum, our results provide a generalization of the neural correlates of value estimation to the affective domain. Furthermore, they suggest that a careful parcelation of striatal structures should be performed in future studies.

The participants in the present work included 55 men and two women. The unbalanced number concerning gender does not allow for the analysis of gender-related variables. In any case, the two females were not outliers and did not influence the RFX analysis.

## Conclusion

Here we extended the value estimation conceptual framework to the social domain, in particular to ingroup belongingness motives. Our work provides evidence for a unique role of the ventral caudate and its association with the left anterior insula in this uniquely human form of goal-oriented decision making involving estimation of value. Whole-brain analysis ensured unbiased identification of these regions, and a role for the nucleus accumbens was only found in ROI-based analysis. In this context, vmPFC does not seem to be directly involved in the estimation of the value magnitude of the affective cues described here, triggered by ingroup belongingness motives. The potential relevance of the functional association between these structures had been highlighted by a recent meta-analysis (Pauli et al., 2016). In conclusion, our work shows that these regions are specifically involved in the computation of the emotional value and reward magnitude expectation in contexts involving passion-related effort, a very special form of cost-benefit decision making involving social and affective values.

## Data Availability Statement

The datasets generated for this study are available on request to the corresponding author.

## Ethics Statement

The studies involving human participants were reviewed and approved by Comissão de Ética da Universidade de Coimbra. The participants provided their written informed consent to participate in this study.

## Author Contributions

ID and MC-B conceived and designed the experiment. RC, SB-C, and ID recruited the participants. SB-C contributed with a personality test and applied the psychological scales. ID and SB-C performed the fMRI experiments. ID and GC analyzed the fMRI data. ID and MC-B wrote the manuscript. All authors contributed to the article and approved the submitted version.

## Conflict of Interest

The authors declare that the research was conducted in the absence of any commercial or financial relationships that could be construed as a potential conflict of interest.
